# Distribution of allele frequencies for genes associated with physical activity and/or physical capacity in a homogenous Norwegian cohort- a cross-sectional study

**DOI:** 10.1186/s12863-020-0813-1

**Published:** 2020-01-23

**Authors:** Sannija Goleva-Fjellet, Anne Mari Bjurholt, Elin H. Kure, Inger Kristin Larsen, Øyvind Støren, Mona Sæbø

**Affiliations:** 1 Department of Natural Sciences and Environmental Health, University of South-Eastern Norway, Gullbringvegen 36, 3800 Bø i, Telemark Norway; 2 Department of Sports, Physical Education and Outdoor Studies, University of South-Eastern Norway, Gullbringvegen 36, 3800 Bø i, Telemark Norway; 3 Department of Cancer Genetics, Institute for Cancer Research, Oslo University Hospital-The Norwegian Radium Hospital, Oslo, Norway; 4 Department of Registration, Cancer Registry Of Norway, Oslo, Norway

**Keywords:** Genes, Polymorphism, *ACTN3*, Physical activity

## Abstract

**Background:**

There are large individual differences in physical activity (PA) behavior as well as trainability of physical capacity. Heritability studies have shown that genes may have as much impact on exercise participation behavior as environmental factors. Genes that favor both trainability and participation may increase the levels of PA. The present study aimed to assess the allele frequencies in genes associated with PA and/or physical capacity, and to see if there is any association between these polymorphisms and self-reported PA levels in a cohort of middle-aged Norwegians of Scandinavian descent (*n* = 831; mean age mean age (± SD) 55.5 ± 3.8 years).

**Results:**

The genotype distributions of the *ACTN3* R577X, *ACE* I/D and *MAOA* uVNTR polymorphisms were similar to other populations of European descent. When comparing the genotype distribution between the low/medium level PA group (LMPA) and high level PA groups (HPA), a significant difference in *ACTN3* 577X allele distribution was found. The X allele frequency was 10% lower in the HPA level group (*P* = 0.006). There were no differences in the genotype distribution of the *ACE* I/D or *MAOA* uVNTR polymorphism. Education and previous participation in sports or outdoor activities was positively associated with the self-reported PA levels (*P* ≤ 0.001).

**Conclusions:**

To the best of our knowledge, this is the first study to report association between *ACTN3* R577X genotype and PA level in middle-aged Scandinavians. Nevertheless, the contribution of a single polymorphism to a complex trait, like PA level, is likely small. Socioeconomic variables, as education and previous participation in sports or outdoor activities, are positively associated with the self-reported PA levels.

## Background

Physical activity (PA) is a complex behavior [1], influenced by both genetic and environmental variables [2–4]. The health effects of PA are well described [5–7], as are the negative consequences of inactivity [8–10]. Insufficient PA levels have been linked to an increased risk of many chronic diseases [11]. Physical inactivity is a modifiable risk factor meaning that increased PA may have a positive effect on several diseases, e.g. diabetes and hypertension [5, 12]. Recommendation for maintaining good health is aerobic PA for a minimum of 150 min per week at moderate intensity or a minimum of 75 min per week at high intensity [10]. Despite strong evidence for genetic influence on PA [1], it is complex and not yet fully understood.

There are large inter-individual differences in PA levels [13] and trainability [14, 15]. Genes influence response to exercise as well as intrinsic behavior like motivation for activity [16, 17]. Thus, genes that favor both trainability and participation may increase the levels of PA [4, 14]. With increased age, heredity may have an even larger impact on exercise participation behavior [18]. Twin studies have shown that up to 62% of PA levels may be explained by genetic factors [4]. However, PA level is affected by the interaction of many genes, most of them with only a small effect each [13].

The two genes most studied in relation to trainability of cardiovascular traits [7, 19], physical function [20] and muscle strength [21] are α-actinin-3 (*ACTN3)* and angiotensin-converting enzyme (*ACE)*. *ACTN3* is a member of the alpha-actin binding protein family. It is predominately expressed in fast twitch muscle fibers [22]. The wild type RR genotype has been reported to be more common among athletes in sprint/power sporting disciplines [23, 24]. The bases for many previous studies have been ethnically highly heterogenous cohorts [25]. Around 18% of the European population are homozygous for the minor allele R577X polymorphism, a premature stop codon [23], with large differences in the minor allele frequency among populations. The frequency of the X allele covaries with the latitude gradient [26]. Absence of the α-actinin-3 protein has been associated with a range of alterations in muscle function [27], including more efficient muscle metabolism [28], increased post-exercise muscle damage and risk of injuries [25]. The R577X polymorphism is one of only two known loss-of-function polymorphisms in humans known to have a selective advantage [27], it is more frequently observed in athletes participating in endurance disciplines [23, 24].

*ACE* codes for the angiotensin-converting enzyme and plays a role in blood pressure regulation [29]. It also influences skeletal muscle metabolism [30] and thus aerobic capacity [31, 32]. Aerobic capacity has been shown to be an important determinant for PA levels [33]. The *ACE* insertion/deletion (I/D) polymorphism is a length polymorphism, where a 287-bp *Alu* repeat is either present or absent [34]. The D allele is mostly associated with sprinting performance ability [35], while the I allele is associated with endurance performance ability [35, 36]. Studies conducted on non-athletes have revealed that the *ACE* I/D polymorphism may also influence responses to strength training [37]. Even though it has been suggested to influence the PA level [38, 39], the results are inconclusive [40].

In addition to genes influencing physiological exercise responses, genes altering PA motivation may also influence the PA level [16]. The dopaminergic system has been a subject to a number of studies on voluntary PA due to its role in reward systems and motor movement, [16, 41–43] and it has been shown to influence the inherent motivation to run in female mice [42].

Monoamine oxidase A (*MAOA*) is one of the genes in the dopaminergic pathways [44] that have been found to influence sedentary behavior [16]. It codes for an enzyme involved in oxidation of neurotransmitters, especially, serotonin, norepinephrine and dopamine [45], and may thus play a role in behavior [42, 44]. The *MAOA* gene is located on the X chromosome and a variable number of tandem repeat sequence upstream from *MAOA* (*MAOA* uVNTR) has been shown to influence the transcription levels of the enzyme [44]. Six alleles have previously been reported [46] with the major *MAOA* allele 3 having lower transcriptional activity (TA) than the other common alleles 3.5 and 4 (high TA alleles) [44, 47] It has therefore been hypothesized that individuals with *MAOA* high TA alleles degrade monoamine neurotransmitters more rapidly ultimately leading to lower PA levels [16]. Also the ACE gene may play a role in the dopaminergic pathways [48, 49], as the renin-angiotensin system, which the enzyme is a part of, and the dopaminergic system interacts [50]. Thus, *ACE* might be involved in the neurobiological regulation of exercise motivation [13, 16]. However, evidence for such relationships is still weak.

Identifying the role of genes and investigating their effect on PA behavior may contribute to further understanding of the large individual differences in PA behavior. Since many studies have been performed on either athletes, well-trained participants or patient groups, they generally have a low number of participants, and seldom represent the general population. In addition, there are large variations in allele frequencies between populations. In order to advance the knowledge, studies on larger and more homogenous cohorts are needed.

The functional *ACTN3* R577X polymorphism has been associated with a range of different exercise and performance related phenotypes, but few studies, if any, have investigated its relation to PA levels. On the other hand, both *ACE* I/D and *MAOA* uVNTR polymorphisms have been studied in relation to PA phenotypes, however, the results have been inconsistent. Therefore, the aim of the present study was to assess *ACTN3*, *ACE* and *MAOA* allele frequencies in a cohort of middle-aged Norwegians of mainly Scandinavian descent, and to investigate any associations between these genes and self-reported PA levels. In addition, the authors wanted to look for associations between socioeconomic variables, such as education and previous participation in sports or outdoor activities, and self-reported PA levels.

## Results

### Demographics

Blood samples and questionnaire data for 416 males and 415 females were available in this study. The mean age of the subjects was 55.5 ± 3.8 years. Participants were slightly overweight, as the mean BMI was 26.1 ± 3.8. However, the BMI in the HPA group was significantly lower than in the LMPA level group (*P* = 0.001; Table 1). The proportion of the cohort with higher education was 24.7%.

**Table 1:** Anthropometric data according to self-reported PA level

	Low/medium PA level			High PA level		
Variable	All (*n* = 215)	Females (*n* = 82)	Males (*n* = 133)	All (*n* = 616)	Females (*n* = 334)	Males (*n* = 282)
Age (y)	55.8 ± 3.8	56.0 ± 4.3	55.6 ± 3.5	55.5 ± 3.7	55.3 ± 3.7	55.6 ± 3.8
Weight (kg)	83.3 ± 15.6	76.6 ± 15.8	87.4 ± 14.0	76.9 ± 12.9	71.0 ± 11.4	83.8 ± 11.1
Height (cm)	174.7 ± 8.1	167.7 ± 5.0	179.1 ± 6.4	172.5 ± 8.6	166.7 ± 5.7	179.4 ± 6.0
BMI (kg/m^2^)	27.3 ± 4.5	27.3 ± 5.5	27.3 ± 3.7	25.7 ± 3.5^*^	25.5 ± 3.8^*^	26.0 ± 3.0^*^

Data are presented as mean ± SD. n- number of subjects; PA- physical activity; BMI- Body Mass Index. ^*^P ≤ 0.009 different from low/medium PA level

### Physical activity data

Of the 831 participants, 25.9 and 74.1% reported LMPA and HPA, respectively. Females reported a significantly higher PA level compared to males (*P* < 0.01). Regular participation in sports or outdoor activities at a younger age was reported to be 51.7%, and participation was higher among males (59.4%) than females (44.0%; *P* < 0.01). Prior participation in sports and outdoor activities was positively associated with reported PA level later in life (*P* < 0.01). Similarly, higher education was positively associated with higher PA levels (*P* < 0.01).

### Genotype and allele frequency distribution for ACE, ACTN3 and MAOA

Out of the 831 participants, 822 were successfully genotyped for the *ACTN3*, 721 for the *MAOA* and 616 for the *ACE* gene. For the *ACE* gene, 24.5% (*n* = 151) were homozygous for the D allele, 52.6% (*n* = 324) were heterozygous, and 22.9% (*n* = 141) were homozygous for the I allele (Table 2). Allele frequencies for the *ACE* gene were 50.8 and 49.2% for the D and I allele, respectively. Genotype distribution for the *ACTN3* gene was 30.8% (*n* = 253), 50.5% (*n* = 415), 18.7% (*n* = 154) for the RR, RX and XX genotype, respectively (Table 2). The frequency for the R allele was 56.0, and 44.0% for the X allele. The observed genotype frequencies were in HWE for all genotypes. Genotype frequencies did not differ significantly between male and female subjects for either *ACTN3* R577X or *ACE* I/D polymorphisms. For the *MAOA* uVNTR polymorphism, five alleles were found: 2, 3, 3.5, 4 and 5 repeat alleles. Frequencies of the rare alleles were as follows: allele 2 was found in one heterozygous female; allele 5 in one hemizygous male and four heterozygous females; allele 3.5 in two hemizygous males and seven heterozygous females (1%). Common allele frequencies were 38.6 and 61.4% for the alleles 3 and 4, respectively. The observed heterozygosity for the common alleles was 42.5%.

**Table 2:** Genotype distribution for the *ACTN3*, *ACE* and *MAOA* gene according to PA level

	Low/medium PA level			High PA level		
Genotype	All (*n* = 215)	Females (*n* = 82)	Males (*n* = 133)	All (*n* = 616)	Females (*n* = 334)	Males (*n* = 282)
*ACTN3*						
Total (n)	214	82	132	608	330	278
RR	23.4%^*^	28.0%	20.5%	33.4%^*^	34.2%	32.4%
RX	58.9%^*^	57.3%	59.8%	47.5%^*^	47.0%	48.2%
XX	17.8%	14.6%	19.7%	19.1%	18.8%	19.4%
*ACE*						
Total (n)	161	65	96	455	250	205
DD	25.5%	29.2%	22.9%	24.2%	26.4%	21.5%
ID	52.8%	46.2%	57.3%	52.5%	52.8%	52.2%
II	21.7%	24.6%	19.8%	23.3%	20.8%	26.3%
*MAOA*						
Total (n)	187	70	117	534	290	244
Low TA^1^	29.4%	14.3%	38.5%	27.7%	15.2%	42.6%
High TA^2^	55.1%	44.3%	61.5%	49.1%	42.1%	57.4%
Heterozygotes^3^	15.5%	41.4%	–	23.2%	42.8%	–

Data are presented as frequency and percentages. n- number of subjects, TA- transcriptional activity genotype. ^1^3-repeat allele male carriers and female homozygotes; ^2^3.5- or 4-repeat allele male carriers and female homozygotes or heterozygotes; ^3^females only; ^*^P < 0.01 difference in *ACTN3* genotype distribution between low/medium and high PA level

**Fig. 1:**
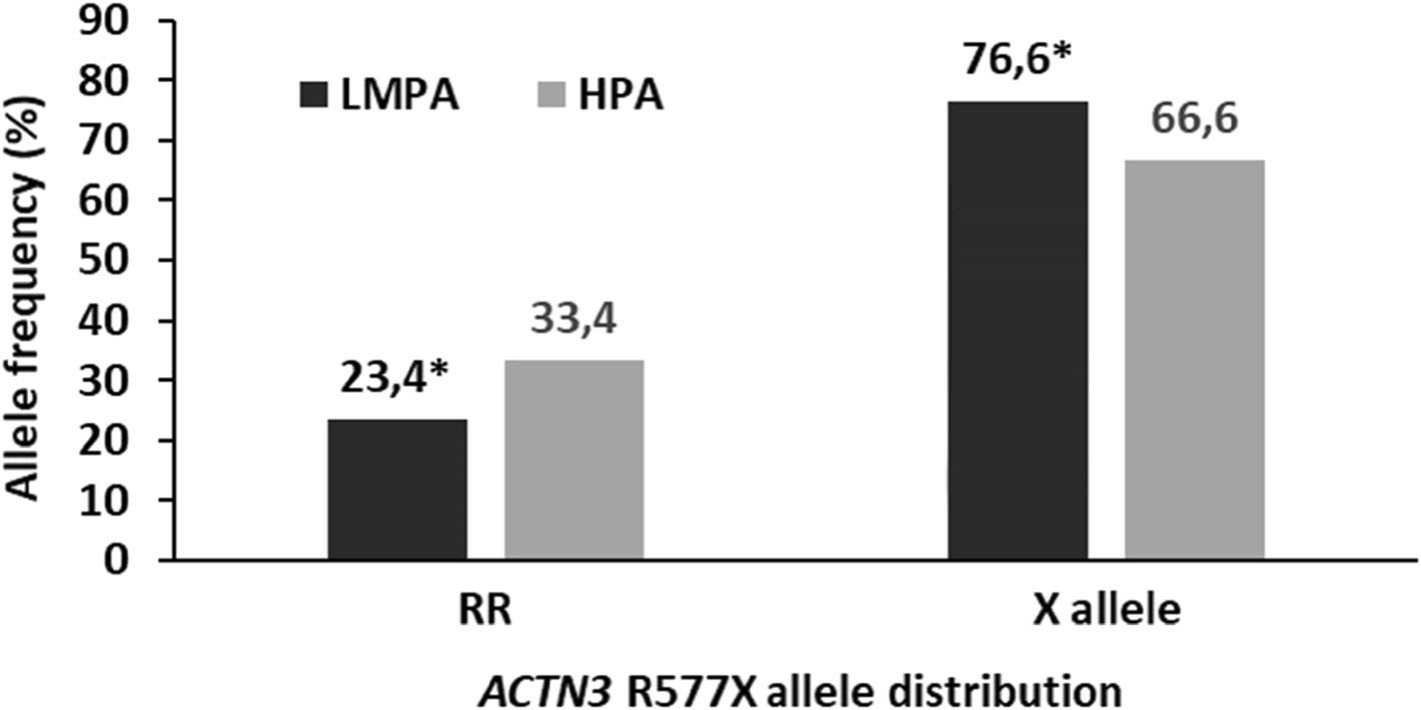
*ACTN3* R577X allele distribution differences between the low/medium and high physical activity groups. *significantly different from the high PA level group (*P* = 0.006). LMPA-low/medium physical activity group; HPA- high physical activity group

The *ACTN3* X allele frequency was 10.0% lower in individuals with HPA than those with LMPA level (*P* = 0.006, Fig. 1). When stratified by sex, significant difference in the X allele was only seen in males (*P* = 0.013).

**Fig. 2:**
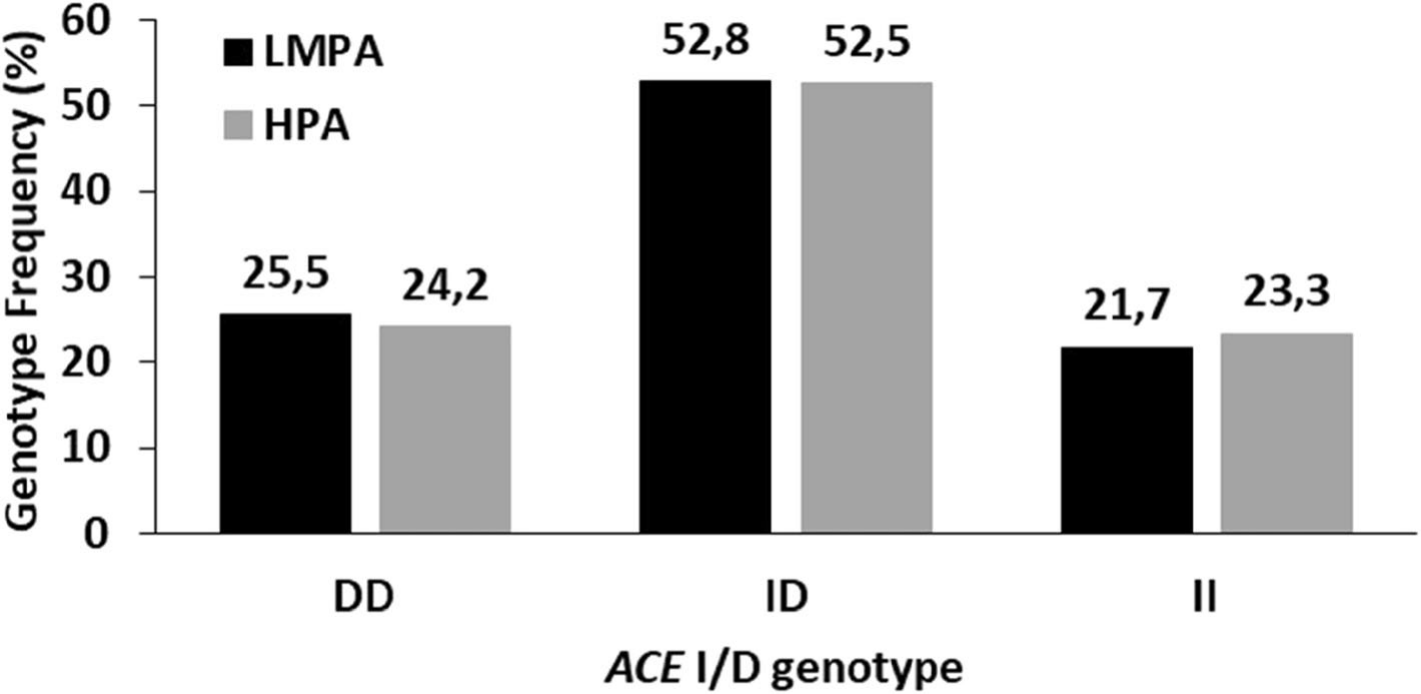
*ACE* I/D genotype distribution in the low/medium and high physical activity groups. LMPA-low/medium physical activity group; HPA- high physical activity group

**Fig. 3:**
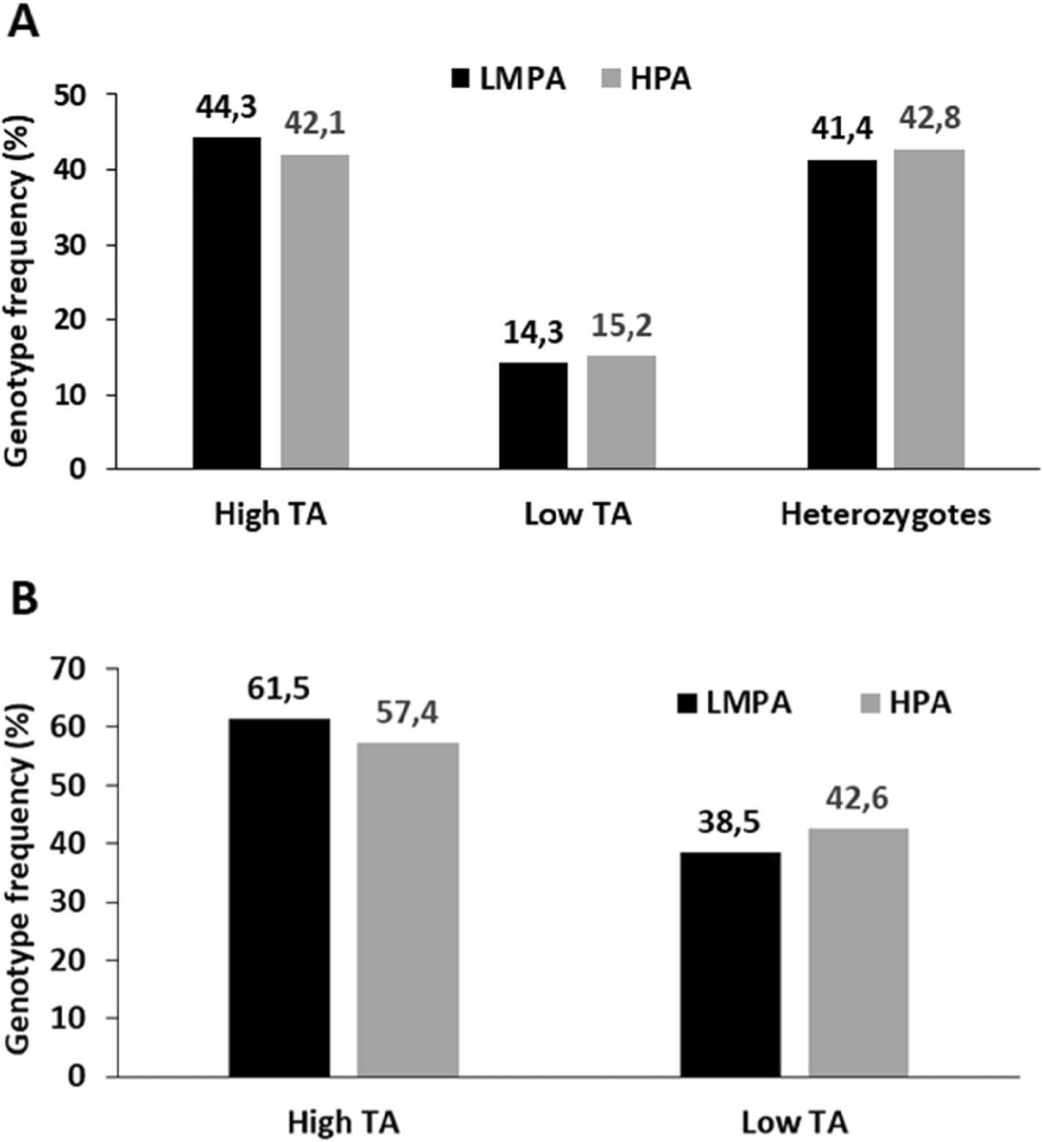
*MAOA* uVNTR genotype distribution in the low/medium and high physical activity groups among female (**a**) and male (**b**) participants. **a** TA- transcriptional activity genotype. Low TA represents 3-repeat allele homozygotes; High TA represents 3.5- or 4-repeat allele homozygotes and heterozygotes carrying one of each high TA alleles; heterozygotes- carriers of one low TA and one high TA alleles. **b** TA- transcriptional activity genotype. Low TA represents 3-repeat allele hemizygotes; High TA represents 3.5- or 4-repeat allele hemizygotes

No associations were found between the ACE I/D (Fig. 2) or MAOA uVNTR (Fig. 3) polymorphisms and the PA level (Table 2).

### Logistic regression models

Gender was found to significantly influence the likelihood of belonging to one of the two PA level groups, i.e. either to LMPA or to HPA level group (*P* < 0.001; Table 3). Males were less likely (OR: 0.47) to belong into the HPA level group compared to females. The BMI was more likely to be lower among subjects in the HPA level group compared to the LMPA level group counterparts (*p* < 0.01; OR = 0.92). Education level showed a statistically significant association (*P* < 0.01) with the PA level. Participants having completed higher education were 2.2 times more likely to belong to the HPA group than the participants with secondary (or lower) education level. Also, subjects who participated in sports/outdoor activities earlier in life were 1.8 times more likely to belong to the HPA level group compared to those that had not (*P* < 0.01).

**Table 3:** Variables entered on step 1: gender, age, BMI, education (2 categories) and participation in sports/outdoor activities earlier in life (2 categories)

Variables included in the equation	B	S.E.	Wald	df	Sig.	Exp(B)	95% C.I.for EXP(B)	
							Lower	Upper
Gender (Male/Female^r.c.^)	−0.753	0.205	13.513	1	**0.000**	0.471	0.315	0.704
Age	−0.035	0.027	1.731	1	0.188	0.965	0.915	1.018
BMI	−0.067	0.026	6.791	1	**0.009**	0.935	0.889	0.983
Education (Higher/Secondary^$r.c.^)	0.805	0.263	9.358	1	**0.002**	2.236	1.335	3.745
Sports earlier in life (Yes/No^r.c.^)	0.599	0.203	8.702	1	**0.003**	1.821	1.223	2.711
Constant	4.080	1.723	5.606	1	0.018	59.173		

Significant values (*p* < 0.05) are indicated in bold; ^r.c^.- reference category; ^$^ - Secondary education or lower

In the second logistic regression model, the genotype data were added to the socioeconomic factors tested previously. Those socioeconomic variables that contributed significantly to the PA level, remained significantly associated in the second model (Table 4). In addition, the *ACTN3* R577X polymorphism was significantly associated with the PA levels (*P* < 0.01). Subjects with the RX genotype were more likely to belong to the LMPA level group (*P* = 0.001; OR = 0.43) compared to the RR genotype subjects. Neither *ACE* I/D nor *MAOA* uVNTR polymorphism showed a significant association with the PA levels.

**Table 4:** Variables entered on step 2: gender, age, BMI, education (2 categories), participation in sports/outdoor activities earlier in life (2 categories), *ACE* I/D polymorphism (3 categories), *ACTN3* R577X (3 categories) and *MAOA* uVNTR (3 categories)

Variables included in the equation	B	S.E.	Wald	df	Sig.	Exp(B)	95% C.I.for EXP(B)	
							Lower	Upper
Gender (Male/Female^r.c.^)	−0.769	0.253	9.218	1	**0.002**	0.464	0.315	0.704
Age	−0.029	0.027	1.134	1	0.287	0.971	0.915	1.018
BMI	−0.071	0.026	7.252	1	**0.007**	0.932	0.889	0.983
Education (Higher/Secondary^$r.c.^)	0.843	0.267	9.944	1	**0.002**	2.324	1.335	3.745
Sports earlier in life (Yes/No^r.c.^)	0.643	0.207	9.635	1	**0.002**	1.902	1.223	2.711
*ACE* (all genotypes)			0.611	2	0.737			
*ACE* (ID/DD^r.c.^)	0.183	0.248	0.545	1	0.461	1.201	0.738	1.953
*ACE* (II/DD^r.c.^)	0.188	0.297	0.402	1	0.526	1.207	0.675	2.16
*ACTN3* (all genotypes)			11.473	2	**0.003**			
*ACTN3* (XX/RR^r.c.^)	−0.491	0.322	2.325	1	0.127	0.612	0.326	1.15
*ACTN3* (RX/RR^r.c.^)	−0.843	0.251	11.268	1	**0.001**	0.430	0.263	0.704
*MAOA* (all genotypes)			0.777	2	0.678			
*MAOA* (Low TA/High TA^r.c.^)	0.211	0.242	0.758	1	0.384	1.235	0.768	1.987
*MAOA* (Heterozygotes/High TA^r.c.^)	0.089	0.317	0.079	1	0.778	1.093	0.588	2.035
Constant	4.816	1.719	7.852	1	0.005	123.475		

Significant values (*p* < 0.05) are indicated in bold; ^r.c^.- reference category; ^$^ - Secondary education or lower

## Discussion

Allele frequencies for the *ACTN3* R577X and the *ACE* I/D polymorphisms has been reported to be highly variable between different ethnic groups, and the X allele has previously been reported to be much more common in Japanese (55%) [51] than it is in Kenyans (9%) [52]. Prevalence of the *ACTN3* X allele in the present study was similar to populations of European descent, with around 45% of individuals being carriers of the minor allele [23].

The frequency distributions for the *ACE* alleles in the present study are also in line with other populations of European descent [30] i.e. 25–50% - 25% for the II, ID and DD alleles respectively. For the ACE I/D polymorphism, distribution of the D allele ranges from 10% for the D allele in Samoans [53], to around 60% in African Americans [54]. Genotype frequencies in other studies on Norwegian subjects were comparable with the frequencies in the present study [55, 56]. However, in one of the studies, the DD genotype was reported to be more prevalent than in other European populations. This is likely due to the preferential amplification of the D allele in heterozygotes, leading to mistyping of some heterozygotes as homozygotes for D allele [57]. That particular study did not used the insertion-specific primers to avoid mistyping of the ID genotype. The large variations in allele frequencies among different ethnicities is important to take into account when doing candidate gene studies [58]. Thus, analyzing homogenous cohorts [54], or accounting for the stratification [58] may improve study power. For the *MAOA* uVNTR polymorphism the allele frequencies were similar to those previously observed in Europeans [44, 47].

The present study found differences in the *ACTN3* R577X allele distribution between the LMPA and the HPA level group. The logistic regression model indicated that the RX allele carriers were more likely to belong to the LMPA level group compared to the RR counterparts. Furthermore, individuals reporting HPA demonstrated higher frequency of the R allele compared to those reporting LMPA. Interestingly, when analyzed by gender, only males demonstrated significant differences in allele distributions between the two PA level groups. Although the authors have not found other studies reporting a relationship between the *ACTN3* gene and PA levels in the general public, it has been suggested to be a potential candidate gene for PA behavior in mice models [13]. The *ACTN3* R577X polymorphism has been linked to trainability of various cardiovascular traits [7, 19] which could, in turn, influence PA behavior [4]. Furthermore, the polymorphism has been associated with traits like sarcopenia [59], muscle function [51] and strength [60]. Previous research indicates that these may be important correlates of PA phenotypes [61–64]. Animal studies have shown changes in signaling and metabolism, among other things [27], which might help to explain the differences in phenotypes between the different genotypes.

Only few studies have been performed on *ACE* I/D polymorphism and PA levels in adults. Similarly to Fuentes et al. [40], the present study could not find any relationship between the *ACE* I/D polymorphism and PA levels, although some previous studies have found an association [38, 39].

The *MAOA* uVNTR polymorphism has a potential to be a candidate gene for influencing PA due to the phenotypic differences in transcriptional activity. Higher TA is expected to lead to higher monoamine oxidase activity and thus lower levels of monoamine neurotransmitters [44]. This, in turn, may lead to different PA level phenotypes [16]. However, the present study could not confirm the findings of Good et al. [16] who observed higher levels of PA in girls homozygous for the low TA allele compared to the high TA allele counterparts. It is still unclear whether the high and low TA alleles influence the monoamine oxidase A enzyme activity in the brain, as Fowler et.al [65]. was not able to measure significant enzyme activity differences in brains of healthy male participants.

Results from the present study showed a strong association between the present PA level and PA at younger ages (*P* < 0.01). Education also correlated with PA levels in the present study (P < 0.01). Both education level [66, 67], and PA activity level at younger ages [66, 68], have in previous studies been shown to correlate positively with present PA levels.

A large proportion of the present cohort reported high PA level (74%). This could be, in part, due to the use of questionnaires as a method for determining the PA levels in the present study. Questionnaire-based methods have been shown to over or underestimate PA behavior compared to the objectively measured PA. The subjective nature of questionnaires may explain the large variation in PA levels observed between different studies [69]. Nevertheless, due to cost efficiency, questionnaires are often used in large epidemiologic studies, including genetic studies [1]. Another questionnaire-based study on a large European cohort of older subjects reported relatively low proportion of participants with no vigorous/moderate physical activity. The overall prevalence of inactivity in the cohort was reported to be 12.5%, with the Scandinavian countries demonstrating some of the lowest rates, i.e. 4.9 and 7.5% in Sweden and Denmark, respectively [64].

The present cohort was randomly drawn from NORCCAP, a homogenous Scandinavian population study with a high attendance rate [70]. The data from the questionnaires allowed the authors to map the ethnicity of the participants. Out of the 831 participants, only two did not have grandparents of Scandinavian descent or lacked the information about ethnicity. The remaining 829 (99,8%) had grandparents of Scandinavian descent. According to Marchini, Cardon [71], a well-known problem with genetic association studies is the undetected population structure such as heterogeneous ethnicity. In the present study, the material may be regarded as ethnically homogenous based on the results from the questionnaires. This can be regarded as one of the strengths of the present study, as a homogenous cohort reduces the chances of both false positive results and failures to detect genuine associations [71]. A further stratification based on ethnicity was therefore not necessary or possible.

Although study population was overweight, the differences in the BMI between the LMPA and HPA groups may also indicate that the self-reported PA levels are reliable [72]. Increase in adiposity (BMI) has been reported to be the cause of decrease in PA levels, as opposed to being the consequence of inactivity [43, 73, 74]. The design of the present study would have been strengthened by including direct measurements of the PA levels to validate the questionnaire data [69].

## Conclusions

The present study demonstrates a novel finding that the X allele of the *ACTN3* gene is underrepresented among participants reporting high PA levels. Genotype data from the present study can be used as a control population in future intervention studies on subjects of European descent. Consistently with previous reports, PA levels in adulthood are associated with factors like education and participation in exercise or outdoor activities earlier in life.

## Methods

### Participants

Blood samples and self-reported PA data were available for 831 individuals from the cohort “Kolorektal cancer, Arv og Miljø (KAM)”, a molecular epidemiological study partly based on the screening group of the Norwegian Colorectal Cancer Prevention Study (The NORCCAP study) in the county of Telemark, Norway [70, 75]. The study design, inclusion/exclusion criteria, participation rates and other relevant information about the NORCCAP study is described in Bretthauer, Gondal [70]. The study was approved by the Regional Medical Ethics Committee of South-Eastern Norway and the Data Inspectorate (REK 3087, S-98052 and S-98190), and is registered in Clinical Trials [76] with the identifier NCT00119912. All procedures performed in studies involving human participants were in accordance with the ethical standards of the institutional and/or national research committee, and with the 1964 Helsinki declaration and its later amendments or comparable ethical standards. Informed written consent was obtained from all individual participants included in the study. Only control subjects (polyp free or polyps with mild grade dysplasia) were included in the present study. Socioeconomic data, including education data, were available for all the subjects. Characteristics of the participants are presented in Table 1.

### Assessment of PA level

PA data was obtained by questionnaire used in the KAM study [75]. The following questions had graded responses on weekly frequency of the activity: “In the last five years, have you walked or bicycled to or from work?”, “Do you hike (cross country)?”, “How often do you exercise for at least 20 minutes?”. Questions “If you exercise, do you perspire?” and “Were you regularly participating in sports or outdoor activities at a younger age?” were dichotomous.

Since the demand for energy differs between various types of activities [77, 78], the different activities were scaled as for example: Hiking = 1 (used as reference, and representing moderate to vigorous intensity), Walking/bicycling = 0.5 (representing low to moderate intensity), Exercise = 1.5 (representing vigorous intensity). By summing the frequencies per week of the scaled activities, each person achieved an activity score. The American College of Sports Medicine (ACSM) has recommended moderate-intensity cardiorespiratory exercise training for at least 30 min, at least five days per week, or at least 20 min of vigorous-intensity cardiorespiratory exercise training for at least three days per week, or a combination of the two training modalities [79]. The activity score of 3 in the present study thus represents the minimum for accomplishing the ACSM physical activity recommendations. For example, a person performing exercise training of 45 min two times per week, or hiking three times per week, or performing walking or cycling six times per week, will reach the activity score 3. Therefore, participants who achieved a score under 3 were defined as inactive or untrained (possessing LMPA) and those who achieved a score ≥ 3 was defined as active/trained (possessing HPA). These PA level groups were assessed for any associations with the ACE I/D, ACTN3 R577X and MAOA uVNTR genotypes, as well as for relationships with gender, education level and previous participation in sports or outdoor activities.

### DNA collection and genotyping

The genomic DNA was extracted from venous EDTA blood stored at -20 °C by using a salting out procedure [80] with minor modifications [75].

The *ACE* I/D polymorphism was genotyped using the Eppendorf Mastercycler Gradient (Eppendorf AG, Germany). Each reaction mixture of 25.5 μl contained 2% DMSO, 1 x PCR buffer, 0.2 mM dNTP, 2 mM MgCl, 0.2 pmol/μl of each primer, 0.5 U/μl Taq polymerase, and 1 μl of DNA (~ 100 ng). Forward and reverse primer were 5′-CTGGAGACCACTCCCATCCTTTCT-3′ and 5′-GATGTGGCCAT-CACATTCGTCAGAT-3′, respectively [34]. Initial denaturation at 95 °C for 3 min was followed by 30 cycles of denaturation (95 °C; 15 s), hybridization (53 °C; 45 s) and extension (72 °C; 30 s). After final elongation (72 °C; 5 min) the PCR products were stored at 4 °C. These were separated by 6% polyacrylamide gel electrophoresis (PAGE) for 30 min at 150 V, and resulted in three possible outcomes (DD, ID and II).

The ACE I allele is often weakly amplified in heterozygotes. Samples with the DD genotype were therefore re-analyzed by using a different PCR reaction in order to avoid mistyping of heterozygotes as DD. Insertion specific forward primer 5′-TTTGAGACGGAGTCTCGCTC-3′ and standard reverse primer [57] were used. Each reaction of 25.0 μl contained 12.5 μl AmpliTaq Gold® PCR Master Mix (Thermo Fisher Scientific, Inc.; MA, USA), 5% DMSO, 0.2 pmol/μl of each primer, and ~ 100 ng template DNA. PCR reaction conditions were as follows: initial denaturation at 95 °C for 3 min followed by 30 cycles of denaturation (92 °C), hybridization (61 °C) and extension (72 °C) for 1 min each. After final elongation (72 °C; 7 min) the PCR products were visualized by 6% PAGE. Insertion specific PCR reaction yielded a 408 bp long DNA fragment in carriers of the I-allele and no PCR product in DD subjects. 215 (25.8%) samples yielded no genotype results ever after repeated PCR runs.

Genotyping of the *ACTN3* R577X polymorphism was carried out with TaqMan® SNP Genotyping Assay, assay ID C____590093_1 (Applied Biosystems®, CA, USA) on the StepOnePlus™ Real-Time PCR System (Applied Biosystems®, CA, USA). Genotype calling was performed by StepOne Software v2.0. Each 15 μl reaction genotyping mixture contained 8.44 μl Genotyping Master Mix, 0.42 μl Assay mix (40x), 6.33 μl distilled H_2_0 and ~ 150 ng of DNA template. Reaction conditions were as follows: 30 s at 60 °C was followed by initial denaturation stage for 10 min at 95 °C; denaturation at 95 °C for 15 s followed by annealing at 60 °C for 1 min in cycling stage with 40 cycles altogether; finally post read temperature was kept at 60 °C for 30 s. Nine samples (1.1%) yielded no genotype results.

The *MAOA* promoter polymorphism was amplified by PCR followed by capillary electrophoresis on an Applied Biosystems 3130xl genetic analyzer using GeneMapper® (Applied Biosystems®, CA, USA) Software 5. Each 15 μl reaction contained 5% DMSO, 1x PCR buffer, 0.2 mM dNTP, 2.5 mM MgCl, 0.4 mM of each primer, 1 U/μl Taq polymerase. The PCR conditions were as follows: initial denaturation at 95 °C (2 min) followed by 35 cycles of denaturation at 95 °C (1 min), annealing at 55.5 °C (1 min) and elongation at 72 °C (2 min), and a final elongation at 72 °C (5 min). Primer sequences have been described earlier [44], and were as follows: a FAM labeled forward primer 5′-ACAGCCTGACCGTGGAGAAG-3′ and a reverse primer 5′-GAACGGACGCTCCATTCGGA-3′. 110 (13.2%) samples yielded no results even after repeated PCR run.

In order to check for reproducibility for *ACE* I/D and *MAOA* uVNTR polymorphisms, approximately 10% of the samples were re-analyzed. In addition, those samples that yielded inconclusive results were re-run. They were excluded from further data analysis if the samples did not yield any genotype or if they remained inconclusive. For *ACTN3* R577X, all samples were run as duplicates.

### Statistical analysis

The material was tested for normality and corrected for multiple testing (Bonferroni method), where appropriate. Association between the BMI and PA level groups was analyzed by using two-tailed independent sample t-test. Pearson’s Chi-square test (χ^2^) was applied to test for the Hardy-Weinberg equilibrium (HWE) for the *ACTN3* R577X, *ACE* I/D genotype, and the differences in categorical variables, including genotype and allelic frequencies between the PA level groups. *MAOA* genotypes were analyzed by dividing genotypes into groups, based on the TA of the alleles [44, 47]. Males carrying the 3-repeat allele and homozygous females for the 3-repeat allele were grouped into low TA, while males carrying either 3.5- or 4-repeat alleles were grouped into high TA group. Similarly, females homozygous for either 3.5 or 4-repeat-alleles and females heterozygous for 3.5 or 4-repeat-alleles were grouped into high TA group. Heterozygous females carrying one 3-repeat and either 3.5- or 4-repeat allele were placed into the heterozygous group. Individuals carrying the rare alleles were excluded from the analysis.

To test the contribution of socioeconomic factors (Gender, Age, BMI, Education, Participation in sports/outdoor activities earlier in life) and genetic variables (*ACTN3* R577X, *ACE* I/D and *MAOA* uVNTR genotypes) to the PA level, binomial logistic regression was used. For this purpose, two models were analyzed: 1. socioeconomic factors only; 2. socioeconomic and genotype data together. Odds ratio (OR) were calculated for the significant associations in the logistic regression. Significance was set at 0.05 for all tests. Results are presented as mean ± SD. All statistical analysis was performed in IBM SPSS Statistics, version 25 (Chicago, IL, USA).

## Data Availability

The datasets generated and/or analyzed during the current study are not publicly available due to Norwegian legislation but are available from the corresponding author on reasonable request.

## Abbreviations

*ACE*: Angiotensin-converting enzyme
*ACTN3*: α-actinin-3
BMI: Body mass index
HPA: High physical activity
HWE: Hardy-Weinberg equilibrium
KAM: «Kolorektal cancer, Arv og Miljø» study
LMPA: Low/medium physical activity
*MAOA*: Monoamine oxidase A
PA: Physical activity
PAGE: Polyacrylamide gel electrophoresis
PCR: Polymerase chain reaction
TA: Transcriptional activity
